# Oral Health Nursing Education and Practice Program

**DOI:** 10.1155/2012/149673

**Published:** 2012-05-17

**Authors:** Maria C. Dolce, Judith Haber, Donna Shelley

**Affiliations:** ^1^New York University College of Nursing, 726 Broadway, 10th Floor, New York, NY 10003, USA; ^2^New York University School of Medicine, 227 East 30th Street, 6th Floor, New York, NY 10016, USA

## Abstract

Millions of Americans have unmet oral healthcare needs and profound oral health disparities persist in vulnerable and underserved populations, especially poor children, older adults, and racial and ethnic minorities. Nurses can play a significant role in improving the quality of oral health including access to care with appropriate education and training. The purpose of this paper is to describe New York University College of Nursing's response to this challenge. The *Oral Health Nursing Education and Practice* (OHNEP) program is a national initiative aimed at preparing a nursing workforce with the competencies to prioritize oral disease prevention and health promotion, provide evidence-based oral healthcare in a variety of practice settings, and collaborate in interprofessional teams across the healthcare system. The overarching goal of this national initiative is to create an educational infrastructure for the nursing profession that advances nursing's contribution to reducing oral health disparities across the lifespan.

## 1. Introduction

Over a decade ago, the United States (US) Surgeon General's landmark report, *Oral Health in America*, profiled the poor oral health status of the nation as a “silent epidemic” and linked oral health to overall health and well-being [1]. While overall improvements in oral health have been reported in the US population, millions of Americans have unmet needs related to oral health and profound oral health disparities persist in vulnerable and underserved populations, especially poor children, older adults, and racial and ethnic minorities [1–3]. For example, today, dental caries (tooth decay), an infectious and highly preventable disease, remains a common chronic disease across the life cycle and disproportionately impacts vulnerable and underserved groups [3].

 One of the many barriers to quality oral healthcare includes a lack of attention to oral health by nondental health care professionals (e.g., nurses, pharmacists, physicians, physician assistants) [1–3]. For example, oral health has not been a high priority for nurses in practice [4]. Another barrier is the inadequate education of nondental health care professionals in basic oral health [3]. To address these challenges, the Committee on Oral Health Access to Services recommended the development of a core set of oral health competencies and curricula for nondental health care professionals to enhance their role in oral health promotion and disease prevention [3]. In response to this recommendation, nursing programs will need to prepare graduates with core competencies to identify risk for oral disease, conduct oral examinations, provide oral health information, connect oral health information with diet and lifestyle counseling, and make referrals to dental professionals [3]. There are over 3 million licensed registered nurses including approximately 140,000 nurse practitioners (NP) in the US health care workforce [5]. With adequate education and training in oral health, the nurse workforce has the potential to have a major impact on improving access and quality of oral health care.

 New York University (NYU) College of Nursing is strategically engaged with NYU College of Dentistry in an innovative organizational partnership to advance an interprofessional model for health professions oral-systemic education and practice. The purpose of this paper is to describe the NYU College of Nursing's program on *Oral Health Nursing Education and Practice* (OHNEP), an outgrowth of the NYU College of Nursing and College of Dentistry academic partnership and interprofessional collaborations with colleagues in Pediatrics and Family Medicine. The OHNEP program is a national initiative aimed at preparing the nurse workforce with the competencies to prioritize oral disease prevention and health promotion, provide evidence-based oral health care in a variety of practice settings, and collaborate in interprofessional teams across the health care system to improve access to care and reduce oral health disparities.

## 2. Materials and Methods

### 2.1. Setting the Stage

 The NYU College of Nursing proposed to develop and demonstrate the impact of a replicable model for implementing and disseminating a comprehensive oral health curriculum in nursing programs and integrate oral health best practices in nurse-managed primary care settings throughout the United States. Several landmark reports published in 2011 set the stage for NYU College of Nursing's program to enhance nursing's role in reducing the burden of oral disease in America. These reports, *Advancing Oral Health in America* [2], *Improving Access to Oral Health Care for Vulnerable and Underserved Populations* [3], *National Prevention Strategy: America's Plan for Better Health and Wellness* [6], and *Core Competencies for Interprofessional Collaborative Practice* [7], underscored the centrality of the nursing profession in improving oral health outcomes, nurses' role in health promotion and prevention, and the importance of interprofessional education and collaborative practice in improving oral health. In 2011, NYU College of Nursing launched a national initiative, *Oral Health Nursing Education and Practice *(OHNEP), funded by DentaQuest Foundation, Washington Dental Service Foundation, and Connecticut Health Foundation. *Oral Health Nursing Education and Practice* is a constituent of the National Interprofessional Initiative on Oral Health (NIIOH), a consortium of clinicians and funders whose mission is to engage primary care clinicians to partner with dental professionals in providing oral health preventive services and to eliminate dental disease.

### 2.2. Program Aims

 The overarching goal of this national initiative is to create an infrastructure for the nursing profession that advances nursing's contribution in reducing oral health disparities across the lifespan. The OHNEP initiative focuses on the development of a replicable model for integrating oral health in nursing curricula and implementing and disseminating oral health best practices in nurse-managed primary care settings. The specific aims of the OHNEP initiative are to

1. engage national nursing stakeholders representing licensure, accreditation, certification, education, practice, and policy in advancing an action plan and recommendations that will support oral health nursing education, clinical practice, and policy changes,
2. implement a strategy for developing oral health competencies in undergraduate and graduate nursing programs,
3. implement a strategy for integrating best practices in oral health care in registered nurse (RN) and advanced practice nurse (APRN) clinical settings,
4. disseminate these strategies nationally including nursing programs, healthcare organizations, nurse managed primary care settings, and professional nursing organizations.

### 2.3. Program Approach

#### 2.3.1. Aim 1

 Engaging stakeholders and creating a shared vision are critical underpinnings of the program approach. To further this aim, in May 2011 a National Invitational Nursing Summit was convened in Washington, DC, to launch the OHNEP initiative. Over 35 representatives from 25 national nursing and professional organizations responsible for licensure, accreditation, certification, education, practice, and policy participated in the Summit. Summit participants were engaged in discussions about nursing's role in improving oral health and expanding access in the context of interprofessional collaboration. These key nursing stakeholders contributed ideas and strategies for advancing an oral health agenda in nursing.

#### 2.3.2. Aim 2

 To achieve this aim a faculty development train-the-trainer approach was designed to enhance nursing curricula and disseminate best practices in oral health. The train-the-trainer workshop, *Oral Health Nursing Education and Best Practices: Enhancing Faculty Capacities*, was specially tailored to assist faculty with integrating oral health into existing courses in the baccalaureate and graduate nursing programs at NYU College of Nursing. The train-the-trainer workshop was approved by NYU College of Nursing's Center for Continuing Education in Nursing, an accredited provider of continuing nursing education by the American Nurses Credentialing Center's Commission. The purpose of the workshop was to provide faculty with teaching-learning resources to facilitate the integration of oral health into didactic, clinical, and simulation learning environments. The *Smiles for Life: A National Oral Health Curriculum* [8] was presented as a comprehensive, interprofessional curriculum for nurse faculty enrichment and competency development in oral health across the lifespan [9]. At the completion of the train-the-trainer workshop, participants were expected to (a) articulate the importance of oral-systemic health and nursing's call to action, (b) discuss interprofessional education and collaborative practice as a framework for improving oral-systemic health outcomes, (c) describe the comprehensive features of Smiles for Life, and (d) implement a variety of teaching-learning strategies that facilitate the development of nurses' oral health competencies and implementation of oral health best practices across the lifespan.

#### 2.3.3. Aim 3

 An oral health documentation or chart template was developed to prompt nurse practitioner (NP) providers to adhere to best practices in oral health care. The chart system will prompt NP providers to assess risk factors for oral disease, provide brief intervention, and make appropriate referrals. This new office system will be pilot tested in a nurse-managed primary care setting to assess the feasibility and preliminary effect on NP adherence to best practices in oral health promotion and disease prevention.

#### 2.3.4. Aim 4

 Laying the groundwork for an effective dissemination plan required extensive outreach to the executive directors, presidents, and conference planning committees of national nursing organizations. Abstracts for preconference train-the-trainer workshops and concurrent sessions were submitted for consideration at national nursing conferences. The target audiences included nurse faculty, professional development specialists, registered nurses, and advanced practice nurses.

## 3. Results and Discussion

### 3.1. Outcome 1

 A short film, *Expanding Access to Oral Health Care—Nurses Make a Difference* [10], featuring Dr. David Satcher, former US Surgeon General, was produced to vividly depict the burden of oral health in America and NYU College of Nursing's response to the challenge of improving oral health. The purpose of the film was to begin a dialogue and create a shared vision about the role of nurses in improving oral health care and access. The film was first presented at the Summit and was highly acclaimed. Since the Summit, the film has been made widely available on the Internet.

### 3.2. Outcome 2

 An outcome of the Summit was the development of a national nursing action plan. The action plan identified short-, mid-, and long-range nursing strategies to advance a national oral health agenda. Priority areas were outlined and included policy, education, practice, interprofessional partnerships, outreach, and communication.

### 3.3. Outcome 3

 One of the first priorities identified by key nursing stakeholders was to establish a National Nursing Workgroup on Oral Health. A national call for members was issued and resulted in nominations from nursing education, practice, research, and policy. The National Nursing Workgroup on Oral Health was first convened in December 2011. The Workgroup, comprised of 18 members, serves as an expert advisory committee providing input related to nursing's role in advancing a national oral health agenda.

### 3.4. Outcome 4

 To keep local, regional, and national constituents up-to-date regarding OHNEP activities, an electronic newsletter, *Oral Health Matters*, was developed for nurses and other health professionals. The inaugural issue was released in Fall 2011 and has been widely disseminated through print media and the Internet.

### 3.5. Outcome 5

 The Smiles for Life (http://www.smilesforlifeoralhealth.org/) curriculum [8] was promoted as a comprehensive oral health resource for competency development and integrated into faculty development train-the-trainer workshops. The curriculum consists of eight individual courses: (1) The Relationship of Oral to Systemic Health, (2) Child Oral Health, (3) Adult Oral Health, (4) Acute Dental Problems, (5) Oral Health and the Pregnant Patient, (6) Fluoride Varnish, (7) The Oral Examination, and (8) Geriatric Oral Health. The NYU College of Nursing's Center for Continuing Education in Nursing, accredited as a provider of continuing nursing education by the American Nurses Credentialing Center's Commission on Accreditation, approved the Smiles for Life curriculum. Each individual course was approved for 1.0 contact hour and was made available free to individual users.

 The Geriatric Oral Health course was the most recent addition to the curriculum. Prior to the launch of the geriatric course in October 2011, a member of the NYU College of Nursing faculty with specialization as an adult and geriatric nurse practitioner conducted an expert review of the content. The OHNEP project director was invited to serve on the National Association of School Nurses National Oral Health Expert Panel and provided consultation related to the implementation of Smiles for Life curriculum for the professional development of school nurses. To date, the OHNEP initiative including Smiles for Life curriculum has received organizational board recognition from the American Association of Colleges of Nursing, National League for Nursing, National Organization of Nurse Practitioner Faculties, National Association of Pediatric Nurse Practitioners, Association of Faculties of Pediatric Nurse Practitioners, and Gerontological Advanced Practice Nurses Association.

### 3.6. Outcome 6

 The train-the-trainer workshop was piloted at NYU College of Nursing for faculty teaching in the baccalaureate and graduate programs. Four workshops were conducted in the Fall 2011 semester. A total of 24 faculty members and 6 students enrolled in the Master's of Science nursing education program completed the workshop. At the end of the workshop, blank index cards were distributed to the participants. The participants were instructed to identify and indicate on the card at least two teaching-learning strategies or resources that they planned to implement in their curriculum as an outcome of the workshop. Participants completed a program evaluation providing feedback on teaching effectiveness and achievement of learning outcomes. Using a five-point likert rating scale ranging from “strongly disagree” to “strongly agree,” participants were asked to rate the following outcomes: (1) I am able to articulate the importance of oral health and nursing's call to action. (2) I am able to discuss interprofessional education and collaborative practice as a framework for improving oral health outcomes. (3) I am able to describe the comprehensive features of Smiles for Life. (4) I am able to implement a variety of teaching-learning strategies that facilitate integration of oral health nursing education and practice across the lifespan. These data were collected at the end of each train-the-trainer workshop. Evaluation data were analyzed to improve the quality of the workshops in meeting faculty learning needs and expectations. Follow-up surveys with workshop participants are conducted to assess how the training has impacted their courses and curriculum.

### 3.7. Next Steps

 The OHNEP initiative will build upon the outcomes achieved in its first year, and focus on the national dissemination of strategies for developing oral health competencies in undergraduate and graduate nursing education and integrating oral health best practices in RN and APRN clinical settings. The dissemination plan includes the spread of curricular innovations and best practices across nursing programs, healthcare organizations, nurse-managed primary care settings, and professional organizations. Train-the-trainer workshops will be offered to nurse faculty, professional development specialists, and clinicians and will feature the Smiles for Life curriculum as a resource for competency development. Faculty and clinician train-the-trainer sessions to disseminate strategies for enhancing nursing curricula, developing oral health competencies, and implementing best practices will be presented at select national nursing conferences and meetings beginning in 2012. These conferences will include the American Association of Colleges of Nursing, National League for Nursing, National Organization of Nurse Practitioner Faculty, and American Academy of Nurse Practitioners.

 The next phase will also focus on the implementation of oral health best practices in nurse-managed primary care settings. A best practice protocol will be first implemented in the NYU College of Nursing Nurse Practitioner Faculty Practice, with expansion into its Mobile Health Van Program and Diabetes Care—Lifestyle Center for Older Adults. The implementation will be evaluated for effectiveness and disseminated nationally. The NYU College of Nursing's OHNEP initiative will continue to demonstrate the capacity to advance interprofessional education and collaborative practice in oral health. Program activities will be planned around aligning leadership, leveraging information technology, and supporting curricular development.

 To continue the dialogue and momentum that began at the National Nursing Summit, a nursing leadership colloquium will be convened in 2012. Members of the National Nursing Workgroup on Oral Health, along with other nursing stakeholders, will be invited to participate in a nursing leadership colloquium on oral health. The goals of the colloquium will be to align nursing leaders on key priority areas: licensure, accreditation, certification, education, practice, and policy, and to build consensus about nursing's role in an interprofessional agenda to improve oral health. An expected outcome of the nursing leadership colloquium will be individual and collective ownership of strategic actions that advances a national policy agenda to improve oral health. An initial strategy will be to attain formal recognition and support from the Tri-Council of Nursing.

 An important strategy for the dissemination of oral health nursing education and best practices will be the development of a website to serve as the “knowledge center” for faculty development, competency development, and best practices in oral health across the lifespan. The website will provide open access to online curricular resources. The OHNEP website, under the umbrella of NIIOH, will facilitate the dissemination of oral health nursing education and practice resources for nurses and other health professionals, including the Smiles for Life curriculum.

 Curricular development awards will be available to nurse faculty and clinicians to support the development, implementation, and evaluation of oral health instructional resources. Curricular resources will be used for educating nurses in undergraduate- and graduate-level programs and clinical practice settings. The curricular resources will be peer-reviewed, published, and disseminated through the OHNEP website. An example of an oral health instructional resource is the use of a standardized patient case for simulation learning designed to supplement the Smiles for Life curriculum.

## 4. Conclusion

 New York University College of Nursing, leveraging a novel organizational partnership with NYU College of Dentistry, is uniquely positioned to advance innovative models of interprofessional education and collaborative practice that enhance oral health outcomes. The *Oral Health Nursing Education and Practice* initiative has gained tremendous momentum with its focus on faculty and professional development using a train-the-trainer approach. Building on this initial momentum, the next phase will focus on the expansion of professional development train-the-trainer programs nationally and implementation of a strategy for integrating oral health best practices in nurse-managed primary care settings. It is in this context that NYU College of Nursing is poised to develop, implement, and evaluate the effectiveness of strategies that facilitate the dissemination of oral health nursing education and best practices.
